# Supplementary material for: Genetic transformation and CRISPR/Cas12a-mediated gene editing of European beech (Fagus sylvatica L.) employing a transient protoplast system
Source: Commun Biol. 2026 Aug 26;9:1145. doi: 10.1038/s42003-026-10805-9 (PMC13519033; doi:10.1038/s42003-026-10805-9)
Supplement: Supplementary file 1 [file 42003_2026_10805_MOESM1_ESM.pdf]

Supplementary Figures

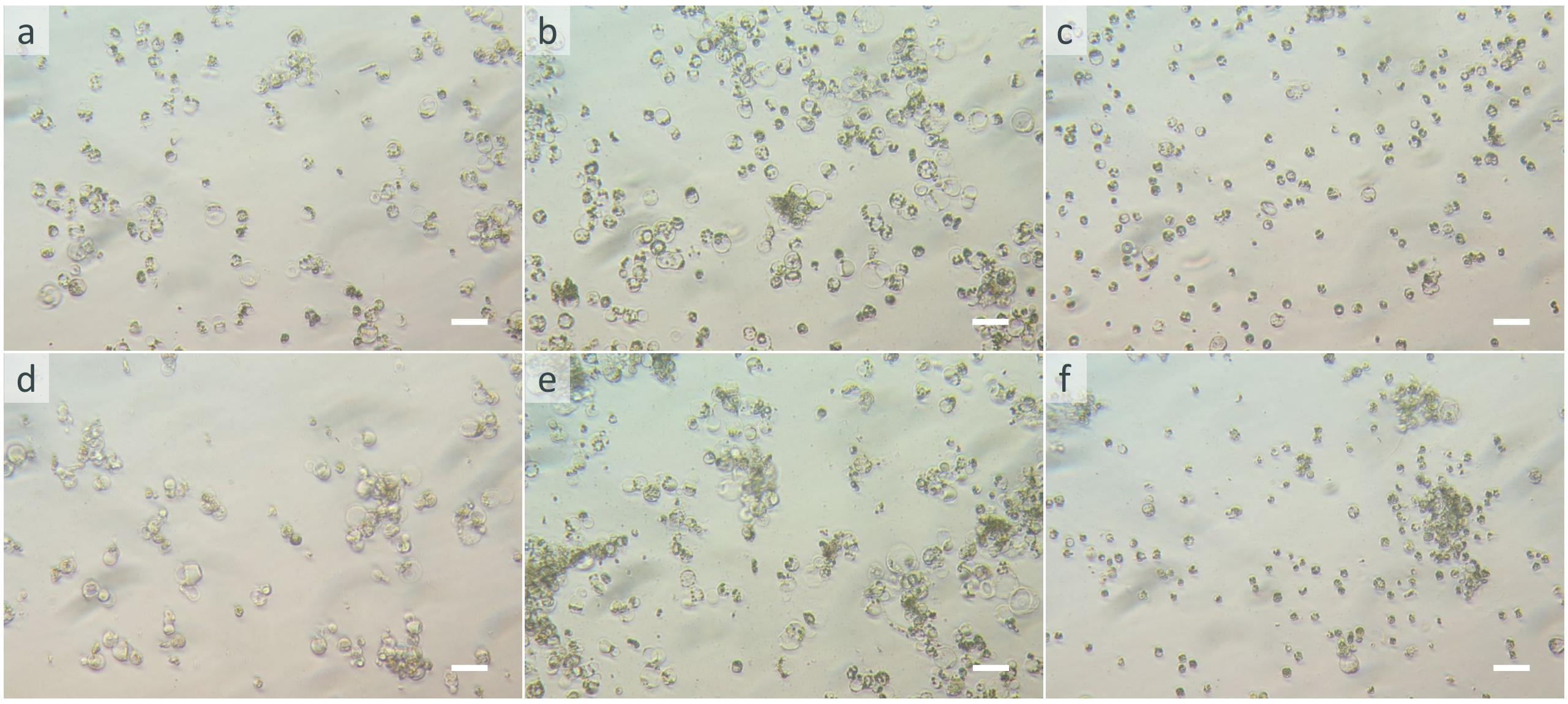

Supplementary Figure 1: Beech protoplast viability after 5 (a-c) and 9 days (d-f) in different media: regeneration medium (a, d), regeneration medium + antibiotics (b, e), regeneration medium + antibiotics + natamycin (c, f). Representative images illustrate effects on survival and morphology of two individual isolations. White scale bars represent 50 µm.

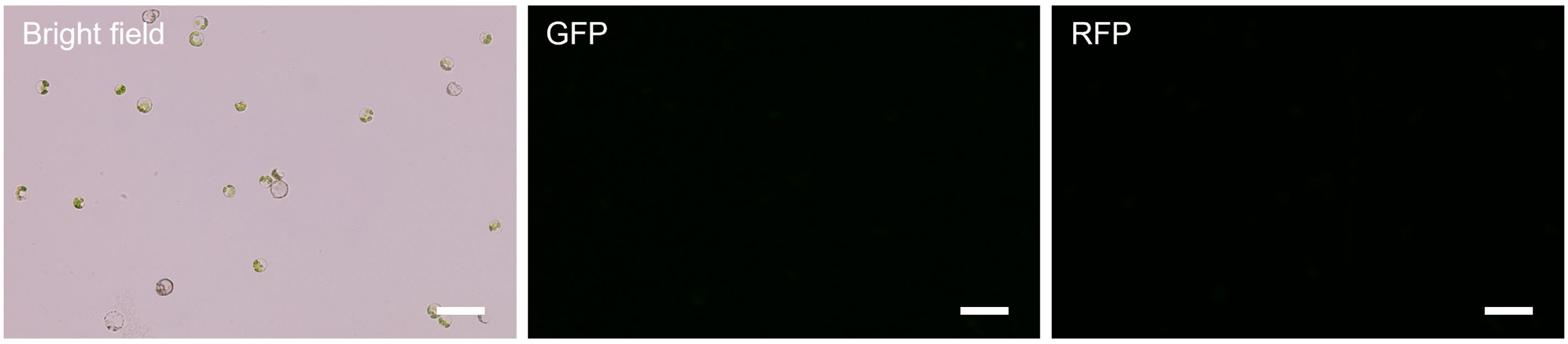

Supplementary Figure 2: Negative control of protoplast transfection for promoter-reporter validation. Bright-field, GFP, and RFP channels of protoplasts transfected without plasmid DNA, imaged under the same conditions as experimental samples. No fluorescence signal was detected in the GFP or RFP channels, confirming the absence of autofluorescence or nonspecific signal. White scale bars represent 50 µm.

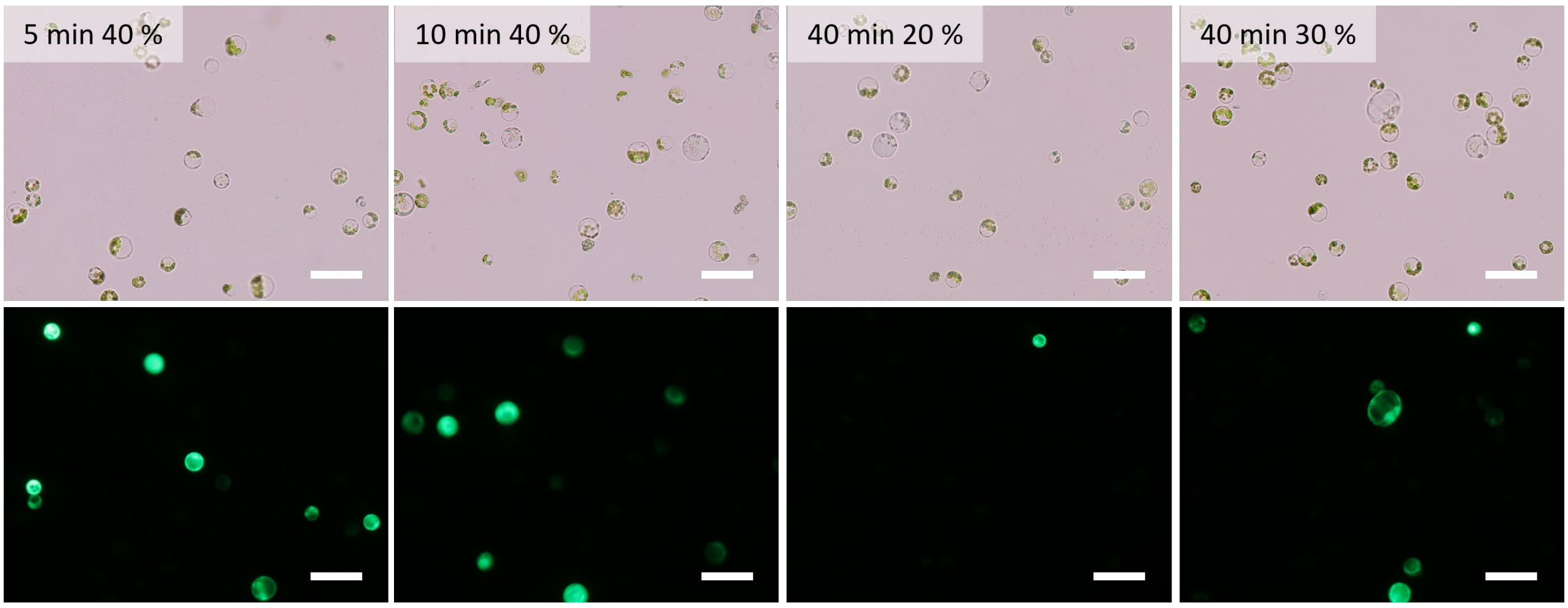

Supplementary Figure 3: Protoplast morphology and mEGFP expression of *F. sylvatica* protoplasts 24 h post-transformation using PEG_{1500}. The panels illustrate the acute effect of varying PEG_{1500} concentration and incubation time on protoplast morphology and subsequent transformation success. Upper panels (bright-field microscopy): protoplast morphology was assessed to evaluate acute PEG-induced cytotoxicity. High vitality was characterized by the maintenance of a spherical shape. Conversely, signs of toxicity are increased morphological damage, including irregularly shaped protoplasts, shrunken cells, and cell debris. The incubation conditions are indicated above each panel (min. and PEG_{1500} concentration [%]). The lower panels (green fluorescence) confirmed the success of transformation (e.g., *mEGFP* expression) under these conditions. White scale bars represent 50 µm.

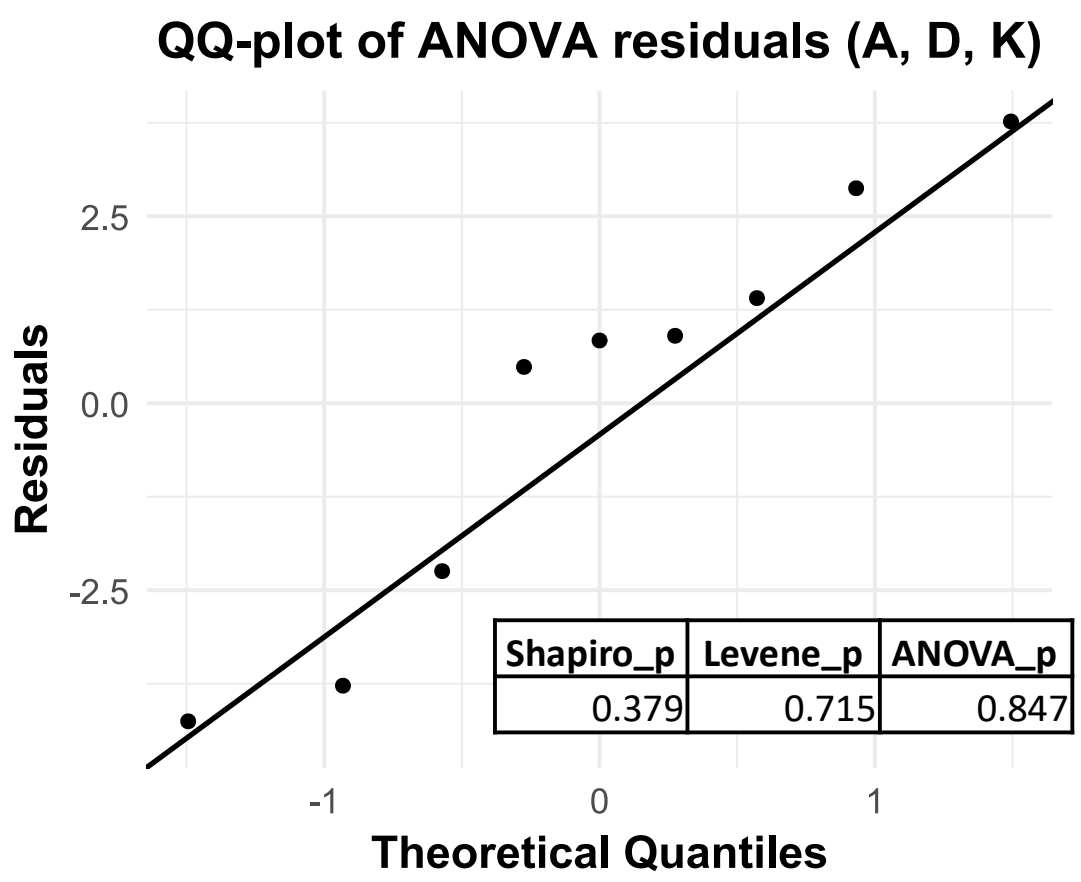

Supplementary Figure 4: QQ-plot of ANOVA residuals for protoplast transformation efficiencies (parameters A, D, and K). The QQ-plot confirms the approximate normal distribution of the residuals, fulfilling a key assumption for the one-way ANOVA. Statistical test results supporting the model's assumptions and the main comparison are presented in the inset table: Shapiro_p (0.379) ≥ 0.05 indicates the residuals are normally distributed. Levene_p (0.715) ≥ 0.05 indicates homogeneity of variances. ANOVA_p (0.847) ≥ 0.05 indicates no significant difference in mean transformation efficiency between parameter sets A, D, and K (n = 3 biological replicates per set).

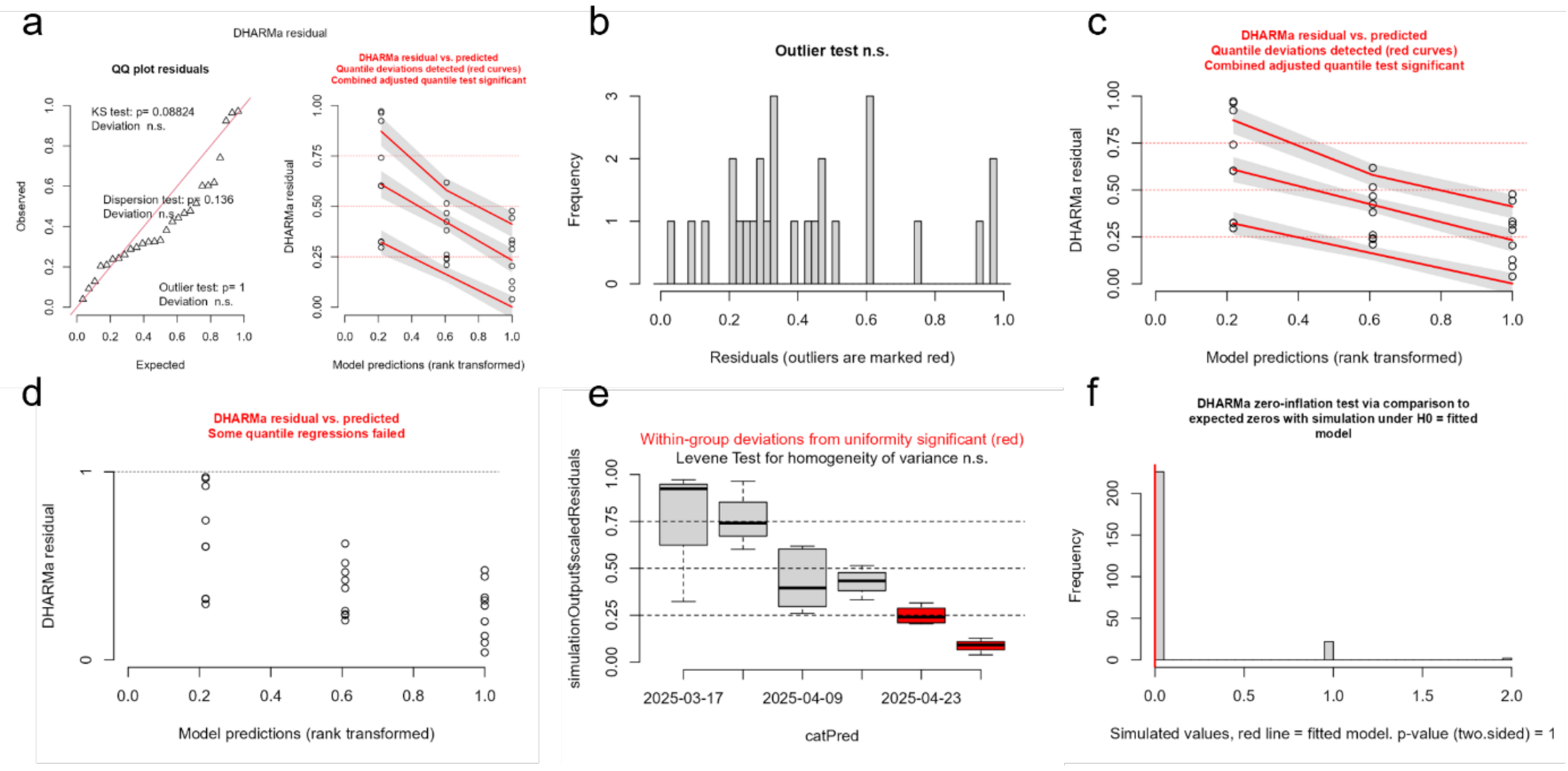

Supplementary Figure 5: Diagnostic plots of GLMM for comparison of transformation parameters over time. The plots show the model diagnostics for the Generalized Linear Mixed Model (GLMM). (a) The QQ-plot indicates a significant deviation from the theoretical distribution, suggesting a lack of fit. (b) The outlier test shows no significant outliers. (c) The quantile residuals plot reveals significant deviations, indicating that the model's assumptions about variance are not fully met. (d) Some quantile regressions failed, further highlighting model limitations. (e) The within-group deviations from uniformity are significant, a potential issue with the nested data structure. (f) The zero-inflation test suggests the model is a good fit for the number of zeros.

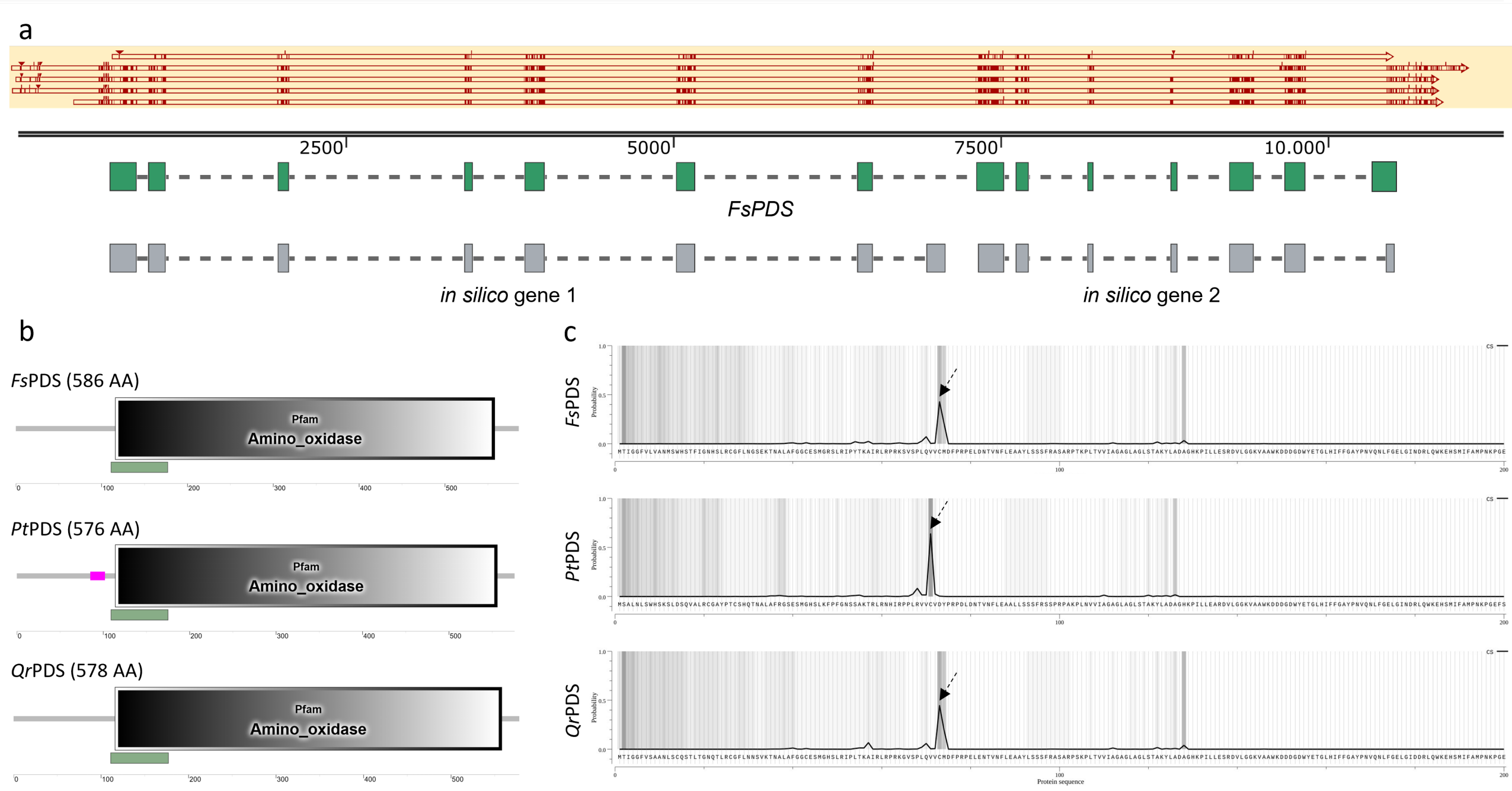

Supplementary Figure 6: Annotation of *F. sylvatica PHYTOENE DESATURASE* (*FsPDS*). (a) Exon–intron boundaries were determined by sequence alignments with *PDS* sequences (shown from top to bottom): CDS from *Populus trichocarpa* (*PtPDS*; Potri.014G148700), mRNA from *Quercus suber* (*QsPDS*; NCBI RefSeq: XM_065777133.1), *Quercus robur* (*QrPDS*; NCBI RefSeq: XM_050436637.1), *Castanea sativa* (*CsPDS*; NCBI RefSeq: XM_075797933.1), and *Quercus lobata* (*QlPDS*; NCBI RefSeq: XM_031066959.1). Additionally, gene models were predicted *in silico* with FGENESH (Softberry). The final gene structure comprises 14 exons. (b) The translated protein sequence was verified by assessing the completeness of PDS-type functional domains (Flavin-containing amine oxidoreductase (Amino-oxidase; PF01593), NAD(P)-binding Rossmann-like domain (PF13450; green bar), low complexity region (pink bar)) using SMART. (c) The presence of a chloroplast transit peptide (arrow) within the PDS proteins was predicted with TargetP-2.0.

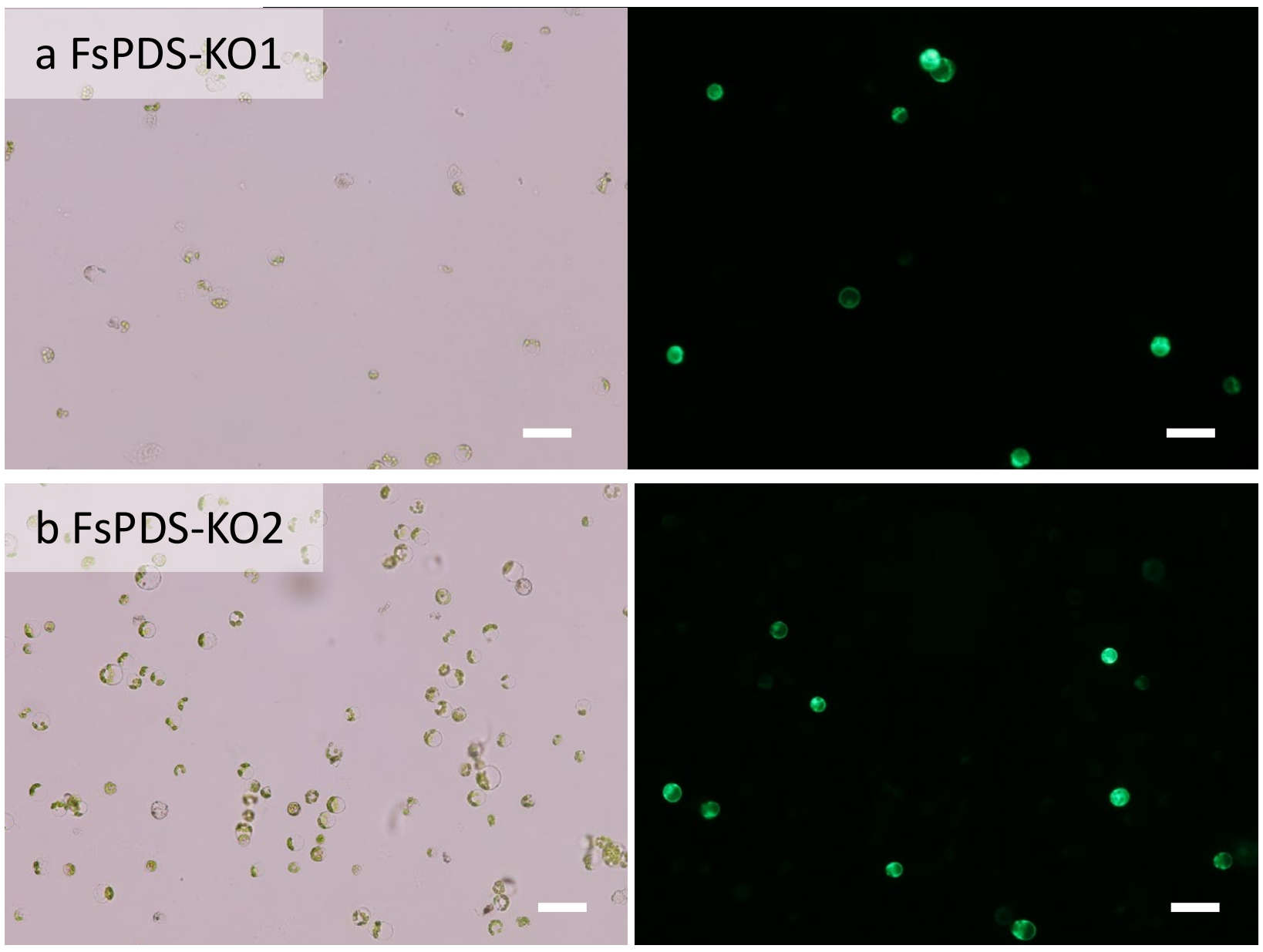

Supplementary Figure 7: *F. sylvatica* protoplasts after transformation with the CRISPR/Cas12a vectors. Representative images of protoplasts 48 hours after transformation with (a) FsPDS-KO1 or (b) FsPDS-KO2. The figure shows the bright-field image (left) and the GFP fluorescence image (right). Green fluorescence confirmed the successful DNA transfer. White scale bars represent 50 µm.

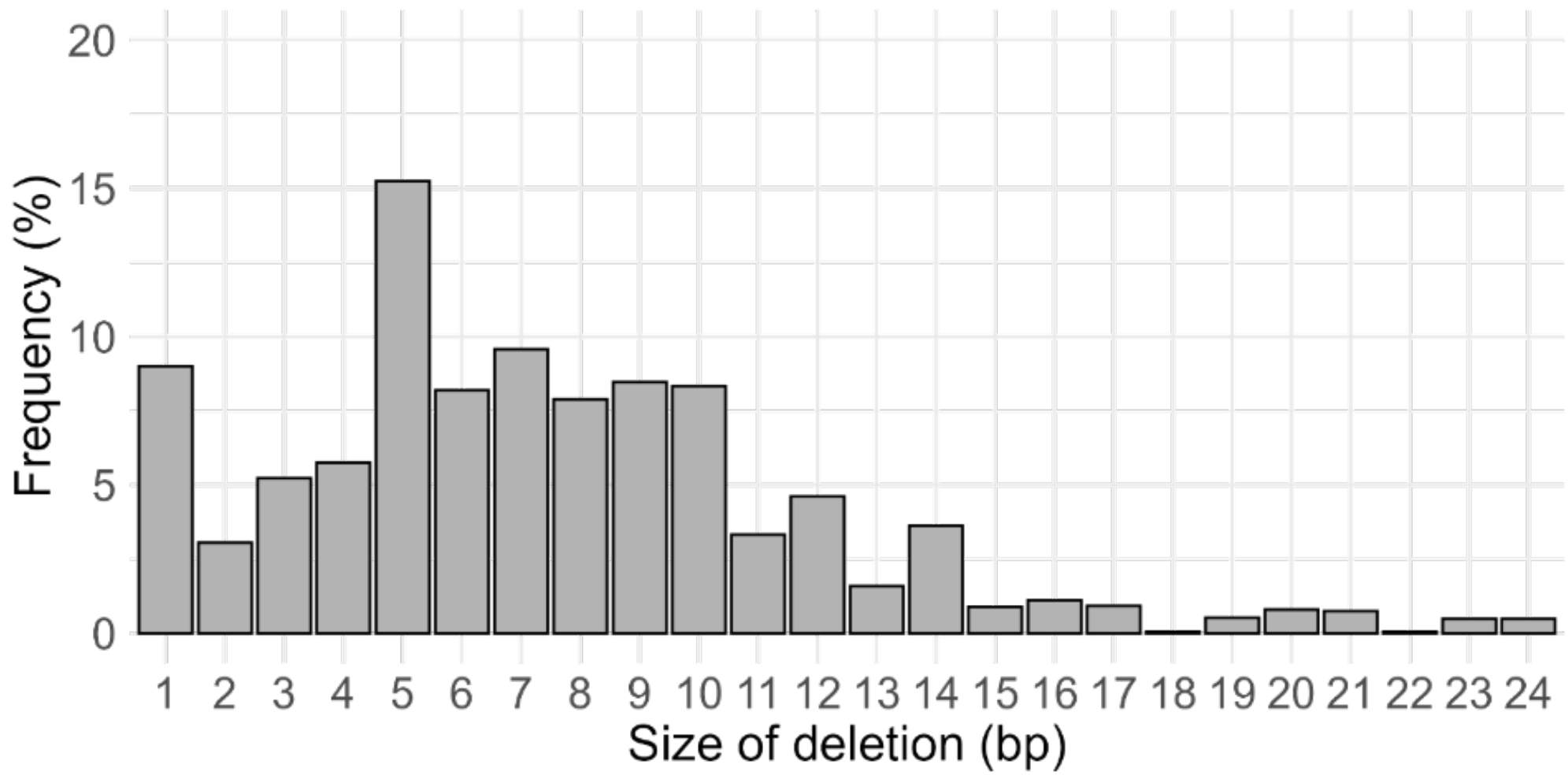

Supplementary Figure 8: Cumulative distribution of deletion sizes induced by tt*Lb*Cas12a across four crRNAs targeting *FsPDS* in *F. sylvatica* protoplasts. Deletion sizes were aggregated across all four crRNA–target combinations. Values represent the summed, normalized fraction of reads for each deletion size. The distribution shows a strong enrichment of small to mid-sized deletions, with the majority of deletion events concentrated between approx. 4 and 10 bp.

Supplementary Tables

Supplementary Table 1: Protoplast isolation from *F. sylvatica* seedling leaves. Yield is expressed relative to the amount of leaf material used—per fresh weight (g^{-1} FW). Protoplast viability was assessed by Evans Blue (EB) or fluorescein diacetate (FDA) staining. The viability rate was calculated as the ratio of blue-colored or green-fluorescent cells to the total number of cells counted (n ≥ 240), respectively. The contamination rate was calculated as the ratio of contaminated wells to all wells (n = 6 or 12). When no contamination rate was assessed (X), no protoplasts remained for culture after the transformation experiments. Values highlighted in gray were not included in the calculation of average protoplast yield or viability, as a precursor protocol or an alternative staining method was used.

Date of isolation	g FW	Protoplasts g^{-1} FW	Staining method	Vitality rate (%)	Contamination rate (%)
28/01/2025	0.81	3.12×10^7	EB	94.17	0.00
17/03/2025	0.68	3.57×10^7	EB	96.66	0.00
25/03/2025	0.55	5.13×10^7	EB	90.04	0.00
27/03/2025	0.61	10.98×10^7	EB	97.74	0.00
09/04/2025	0.81	1.77×10^7	FDA	95.32	0.00
16/04/2025	0.64	14.63×10^7	FDA	95.37	0.00
23/04/2025	0.53	3.90×10^7	FDA	99.19	0.00
28/04/2025	0.60	1.33×10^7	FDA	91.02	X
06/05/2025	0.52	4.16×10^7	FDA	88.76	16.67
25/09/2025	0.52	6.26×10^7	FDA	90.42	0.00
15/10/2025	0.51	3.98×10^7	FDA	96.22	0.00
21/10/2025	0.54	5.56×10^7	FDA	97.16	25.00
24/10/2025	0.53	3.78×10^7	FDA	90.73	X
14/11/2025	0.58	4.29×10^7	FDA	98.12	0.00

Supplementary Table 2: Transformation efficiencies of different promoter reporter constructs (vector) used for *F. sylvatica* protoplast transformation. Efficiency was calculated by the mean of transformation efficiency (% GFP-positive cells relative to total cells) of the three technical replicates. Parameter sets are in accordance with Table 1.

Date of transformation	Parameter set	Vector	Transformation efficiency [%]
17/03/2025	A	35S::*mEGFP*	10.86
17/03/2025	A	*Atu*NOS::*mEGFP*	9.19
17/03/2025	A	*Ha*UBI::*mEGFP*	15.03
17/03/2025	A	*Pc*UBI::*mEGFP*	21.77
24/10/2025	G	35S::*mEGFP*	1.19
24/10/2025	G	*Atu*OCS::*mEGFP*	0.45
24/10/2025	G	*At*UBQ10::*mEGFP*	3.05

Supplementary Table 3: Transformation efficiencies of different parameter sets used for *F. sylvatica* protoplast transformation. Efficiency was calculated by the mean of transformation efficiency (% GFP-positive cells (cells_GFP) relative to total cells (cells_total)) of the three technical replicates. No. and parameter sets are in accordance with Table 1.

Date of transformation	No.	Parameter set	cells_total	cells_GFP	Transformation efficiency [%]
17/03/2025	-	A	378	45	10.86
27/03/2025	I	A	878	84	8.89
27/03/2025	I	P	2029	59	3.28
09/04/2025	II	A	814	34	4.21
09/04/2025	II	B	640	63	9.73
09/04/2025	II	C	1204	16	1.45
09/04/2025	II	D	753	81	10.25
09/04/2025	II	E	493	44	8.91
16/04/2025	III	D	1184	119	9.68
16/04/2025	III	J	1360	3	0.18
16/04/2025	III	K	1086	145	13.30
16/04/2025	III	L	1148	6	0.56
16/04/2025	III	M	908	105	10.79
23/04/2025	IV	D	1072	71	6.60
23/04/2025	IV	K	1167	119	10.02
23/04/2025	IV	N	1253	82	6.49
23/04/2025	IV	O	1022	104	10.45
28/04/2025	-	K	1169	60	5.28
25/09/2025	V	K	805	49	6.15
25/09/2025	V	Q	806	25	3.18
25/09/2025	V	R	684	21	3.14
15/10/2025	VI	K	884	418	46.69
15/10/2025	VI	F	772	454	59.00
15/10/2025	VI	S	907	67	7.42
21/10/2025	VII	F	892	350	39.20
21/10/2025	VII	G	876	366	42.70
21/10/2025	VII	H	1225	282	22.54
21/10/2025	VII	I	1019	261	26.90
21/10/2025	VII	T	1126	139	12.80
21/10/2025	VII	U	1269	97	6.91
21/10/2025	VII	V	1130	143	13.11
24/10/2025	-	G	907	13	1.19
14/11/2025	-	G	1046	88	3.16

Supplementary Table 4: Highest ranked results of BLAST search of *FsPDS* gene against the NCBI database. The *Populus* results are included for comparison.

Species	Accession	Max score	Total score	Query cover (%)	E-value	Identity (%)	Alignment length
Quercus suber	XM_065777133.1	355	1984	13	8e-92	96.3	2426
Quercus robur	XM_050436637.1	355	2523	17	8e-92	96.3	2330
Castanea sativa	XM_075797933.1	350	2528	17	4e-90	95.8	2365
Quercus lobata	XM_031066959.1	346	2368	16	5e-89	95.4	2384
Populus trichocarpa	XM_002321068.4	235	686	6	5e-59	92.2	2195

Supplementary Table 5: Transformation efficiency of beech protoplasts using CRISPR/tt*Lb*Cas12a vectors. Efficiency [%] was quantified as the percentage of green fluorescent cells relative to the total cell count. Mean efficiency is presented for each vector based on three independent technical replicates (Rep I-III; n = 3).

Vector	Transformation efficiency [%]			Transformation efficiency (mean ± SD) [%]
	Rep I	Rep II	Rep III	
FsPDS-KO1	11.20	17.29	10.28	12.92
FsPDS-KO2	8.43	6.53	14.34	9.79

Supplementary Table 6: Deletions detected within ±10 bp of the cut site for *FsPDS*-targeting crRNAs in the wildtype control. Columns indicate the position in the reference sequence, deletion length, deleted bases, number of reads with the deletion, and total reads covering the related reference position in the respective mapping.

crRNA	Reference position	Length [bp]	Reference	Count	Total reads
3_1	174	1	G	4	64006
3_1	177-178	2	CA	3	64006
3_1	180	1	A	1	64006
3_1	192	1	T	5	64006
3_2	192	1	T	5	63899
3_2	199	1	C	4	63899
3_2	200	1	A	1	63899
3_2	206	1	T	1	63899
3_2	208	1	T	3	63899
5_1/5_2	147	1	T	1	81207
5_1/5_2	148	1	A	2	81207
5_1/5_2	151	1	C	1	81207
5_1/5_2	153	1	T	1	81207
5_1/5_2	154	1	G	3	81207
5_1/5_2	156	1	A	1	81207
5_1/5_2	160	1	T	1	81207

Supplementary Table 7: Level-0 (L0) modules used for cloning of L1 promoter-reporter constructs. L0 modules are derived from the MoClo Plant Parts Kit (Addgene #1000000047) and vector backbones from the MoClo Toolkit (Addgene #1000000044). Sequences for additional L0 modules are derived from vectors obtained from H. Puchta, KIT, Karlsruhe, Germany, or DNA Cloning Service (DCS), Hamburg, Germany.

Name	Module	Destination	Source
pICH47811	L1 Backbone vector	All L1 *mEGFP* vectors	Toolkit
pICH47802	L1 Backbone vector	*Pc*Ubi::*turboRF*P	Toolkit
pICH41295	L0 backbone vector	All L0 promoter vectors	Toolkit
pICH41308	L0 backbone vector	L0.C13_*mEGFP*	Toolkit
pICH41276	L0 backbone vector	L0.T12_*At*HSP18.2	Toolkit
pICH51266	CaMV35S+TMVΩ promoter	35S::*mEGFP*	Plant Parts Kit
pICH87633	*Atu*NOS promoter	*Atu*NOS::*mEGFP*	Plant Parts Kit
pICH88103	*Atu*OCS promoter	*Atu*OCS::*mEGFP*	Plant Parts Kit
L0.P13_PcUbi	*Pc*UBI promoter	*Pc*UBI::*turboRFP*	KIT
L0.T12_pea3A	*Pea*3A terminator	*Pc*UBI::*turboRFP*	KIT
L0.P13_AtUbq10	*At*UBQ10 promoter	*At*UBQ10::*mEGFP*	DCS
L0.P13_HaUbi	*Ha*UBI promoter	*Ha*UBI::*mEGFP*	DCS
L0.T12_AtHSP18.2	*At*HSP18.2 terminator	All L1 *mEGFP* vectors	DCS
L0.C13_mEGFP	*mEGFP* CDS	All L1 *mEGFP* vectors	DCS

Supplementary Table 8: Modifications of Level-0 modules for MoClo compatibility.

Module	Position (bp)	Base exchange	RE site	Mutation type
mEGFP CDS	816	G → A	*Bpi*I	silent
*At*UBQ10 promoter	1279	C → G	*Bpi*I	intron
*Ha*Ubi promoter	167	C → A	*Bsa*I	promoter

Supplementary Table 9: Primers used in this study.

Identifier	Primer sequence (5’ → 3’)	Application
3418	gtagaagactgggagtttccttacattctgagc	Cloning of L0.P13_AtUbq10
3419	atggaagactgcctcttcgatctaagattaa	Cloning of L0.P13_AtUbq10
3420	gtagaagactcgaggattttctgggtttgatcg	Cloning of L0.P13_AtUbq10
3421	gtagaagacgtcattctgttaatcagaaaaact	Cloning of L0.P13_AtUbq10
3531	tctaagctctccaaagaccccaacgagaagagg	Cloning of L0.C13_mEGFP
3532	tcgttggggtctttggagagcttagattg	Cloning of L0.C13_mEGFP
3552	gtagaagacgcggaggacaaaacttaagaaagtcacc	Cloning of L0.P13_HaUbi
3553	gtagaagacgtggtagtagaccaacaaatggtatc	Cloning of L0.P13_HaUbi
3556	gtagaagactgaatgggcaagggcgaggaac	Cloning of L0.C13_mEGFP
3557	gtagaagactgaagctcacttgtagagttcatccatgccatgc	Cloning of L0.C13_mEGFP
3558	gtagaagactggctttgtcaaataaatagcttgtgtgc	Cloning of L0.T12_AtHSP18.2
3559	gatgaagactgagcgagctccttatctttaatcatattcc	Cloning of L0.T12_AtHSP18.2
3560	gtagaagactgggagaaaaattacggatatgaatataggcatatc	Cloning of L0.P13_PcUbi
3561	gtagaagactgcattgctgcacatacataacatatcaag	Cloning of L0.P13_PcUbi
3562	gtagaagactggcttcaggcctcccagctttc	Cloning of L0.T12_pea3A
3563	gtgaagactgagcgaagcctatactgtacttaacttgattgc	Cloning of L0.T12_pea3A
3582	gtagaagacggtaccacttacgtaacattaatattccatccg	Cloning of L0.P13_HaUbi
3583	gtagaagacggcattaagacgaagcgaaacaggaagagataag	Cloning of L0.P13_HaUbi
3590	gtagaagacttggaggaaccgcaacgttgaagg	Cloning of L0.P13_AtuNOS
3591	gtagaagacgccattgactctaattggataccgag	Cloning of L0.P13_AtuNOS
3514	ggttgcaaacatgagctggcac	Sequencing *FsPDS* Exon 1-3
3792	ggacaaaaacagagcagcatg	Sequencing *FsPDS* Exon 1-3
3793	tgttccatctcttggttaggg	Sequencing *FsPDS* Exon 4+5
3794	cagggagagtgagggcag	Sequencing *FsPDS* Exon 4+5
ttCas12a_for	gttcaccaactgctacagcc	ttCas12a amplification
ttCas12a_rev	gttcttgttgagggtgttgc	ttCas12a amplification
Ex3_for	agttaaggtactcttgggagatc	Exon 3 amplification
Ex3_rev	gattttctcattgcatcctgtacc	Exon 3 amplification
Ex5_for	ggcaagttggagcttacc	Exon 5 amplification
Ex5_rev	gatgaaatttgtccaccttctacg	Exon 5 amplification

Supplementary Table 10: Edited reference sequences of *FsPDS* exons 3 and 5 used for alignment of amplicon sequencing data.

Exon	*FsPDS* reference sequence
Exon 3	AGTTAAGGTACTCTTGGGAGATCTAATAGTACTAGCTTAATGGATCATTACTTTTTTTTTTTTTTTTTCYT<br>TTTTCTTTTCATTTTCYTTTAATTGTTGCACTATASAGTATAATTTTACAAATTATTGCAGGTTTGGCTGG<br>TTTATCAACTGCCAAGTATTTGGCAGATGCTGGTCACAAACCTATACTATTGGAGTCAAGAGATGTTCTAG<br>GAGGAAAGGTTTTCATGCTGCTCTGTTTTTGTCCCACTTTTTCAATCAGCTGACACGAAACAAGTGCTTGT<br>TTTTCATTTTTGCCTTGCTTGCCACTTTCAAAAACTAGAATTTTTTTTTTTTGGATTGGTAATAAAAATTT<br>CATCAAATGATGAGAGAAATATACATGGGAAACCATTGGTACAGGATGCAATGAGAAAATC
Exon 5	CATTACAAAATTAGGTTGTTTCCTTATTTGATTTTTTTTTGTTGGCAAGTTGGAGCTTACCCAAATGTGCA<br>GAATCTGTTTGGRGAACTTGGTATTAATGATCGGTTGCAATGGAAGGAACATTCTATGATATTTGCGATGC<br>CGAATAAGCCTGGAGAGTTCAGCCGATTTGATTTTCCYGAAGTTCTTCCTGCACCATTAAATGGTATTATA<br>AGAGTAATCACTAGTGCGGCCGCCTGCAGGTCGACCATATGGGAGAGCTCCCAACGCGTTGGATGCATAGC<br>TTGAGTATTCTATAGTGTCACCTAAATAGCTTGGCGTAATC
