# Supplementary material for: Genetic transformation and CRISPR/Cas12a-mediated gene editing of European beech (Fagus sylvatica L.) employing a transient protoplast system
Source: Commun Biol. 2026 Aug 26;9:1145. doi: 10.1038/s42003-026-10805-9 (PMC13519033; doi:10.1038/s42003-026-10805-9)
Supplement: Supplementary file 2 [file 42003_2026_10805_MOESM2_ESM.pdf]

Description of Additional Supplementary Files

Supplementary Data 1: Results ANOVA and GLMM.

Supplementary Data 2: Vector and target sequences.

Supplementary Data 3: Source data for graphs and charts.

Supplementary Data 4: Original microscopic images used in main and supplementary figures.
